# Supplementary material for: Coherent Integration of Organic Gel Polymer Electrolyte and Ambipolar Polyoxometalate Hybrid Nanocomposite Electrode in a Compact High-Performance Supercapacitor
Source: Nanomaterials (Basel). 2022 Feb 1;12(3):514. doi: 10.3390/nano12030514 (PMC8839628; doi:10.3390/nano12030514)
Supplement: Supplementary file 1 [file nanomaterials-12-00514-s001.zip › nanomaterials-1552572-supplementary.pdf]

Coherent Integration of Organic Gel Polymer Electrolyte and Ambipolar Polyoxometalate Hybrid Nanocomposite Electrode in a Compact High-Performance Supercapacitor

Jun-Jie Zhu,[a*] Luis Martinez-Soria[a] and Pedro Gomez-Romero[a,b*]

[a] Catalan Institute of Nanoscience and Nanotechnology (ICN2), CSIC and BIST, Campus UAB, Bellaterra, 08193 Barcelona, Spain.

[b] Consejo Superior de Investigaciones Científicas (CSIC), Spain

* Corresponding author. E-mail address: pedro.gomez@icn2.cat (P. Gomez-Romero), zhu.junjie@icn2.cat (J.J. Zhu) Tel: +34 937373608 (P. Gomez-Romero), Fax: +34 936917640 (P. Gomez-Romero), ICN2, Campus UAB, 08193 Bellaterra (Barcelona) Spain

Supplementary materials 1

Calculation methods for gravimetric capacitance C_m (F g^{-1}), volumetric capacitance C_v(F cm^{-3}), volumetric energy density E_v (Wh cm^{-3}) and power density P_v (W cm^{-3}) are calculated according to the following equation:

For cyclic voltammograms or cyclic polarization:

$$C_{cell} = \frac{\int I(V) \cdot dV}{2v\Delta V} \qquad (S1)$$

For galvanostatic charging and discharging (GCD):

$$C_{cell} = \frac{I \int V(t) \cdot dt}{\Delta V^2} \qquad (S2)$$

$$C_m = \frac{2C_{cell}}{m} \qquad (S3)$$

$$C_v = \frac{2C_{cell}}{V_e} \qquad (S3)$$

$$E_v = \frac{C\Delta V^2}{3600 \times 2V_{total}} \qquad (S4)$$

$$P_v = \frac{I\Delta V}{V_{total}} \qquad (S5)$$

Where I is the discharge current of GCD, C_{cell} is the capacitance of the device, ΔV is voltage window, v is the scan rate of CVs, V_e is the volume (cm^3) of the electrode after subtracting AI foil, m is the mass of active materials on one electrode, V_{cell} is the volume of the two electrode (including the current collector) and the separator.

The modified restricted Warburg diffusion element (W_s) is defined by the following equation.

$$Z(f) = \frac{R\coth\,(\tau i 2\pi f)^{\frac{a}{2}}}{(\tau i 2\pi f)^{\frac{a}{2}}} \qquad (S6)$$

where R is the diffusion impedance (W_s-R), τ is the diffusion time (W_s-T), and a is the exponential factor (W_s-P). τ relates to the effective diffusion thickness (L) and the diffusion coefficient (D), as show below:

$$\tau = \frac{L^2}{D} \qquad (S7)$$

Supplementary materials 2

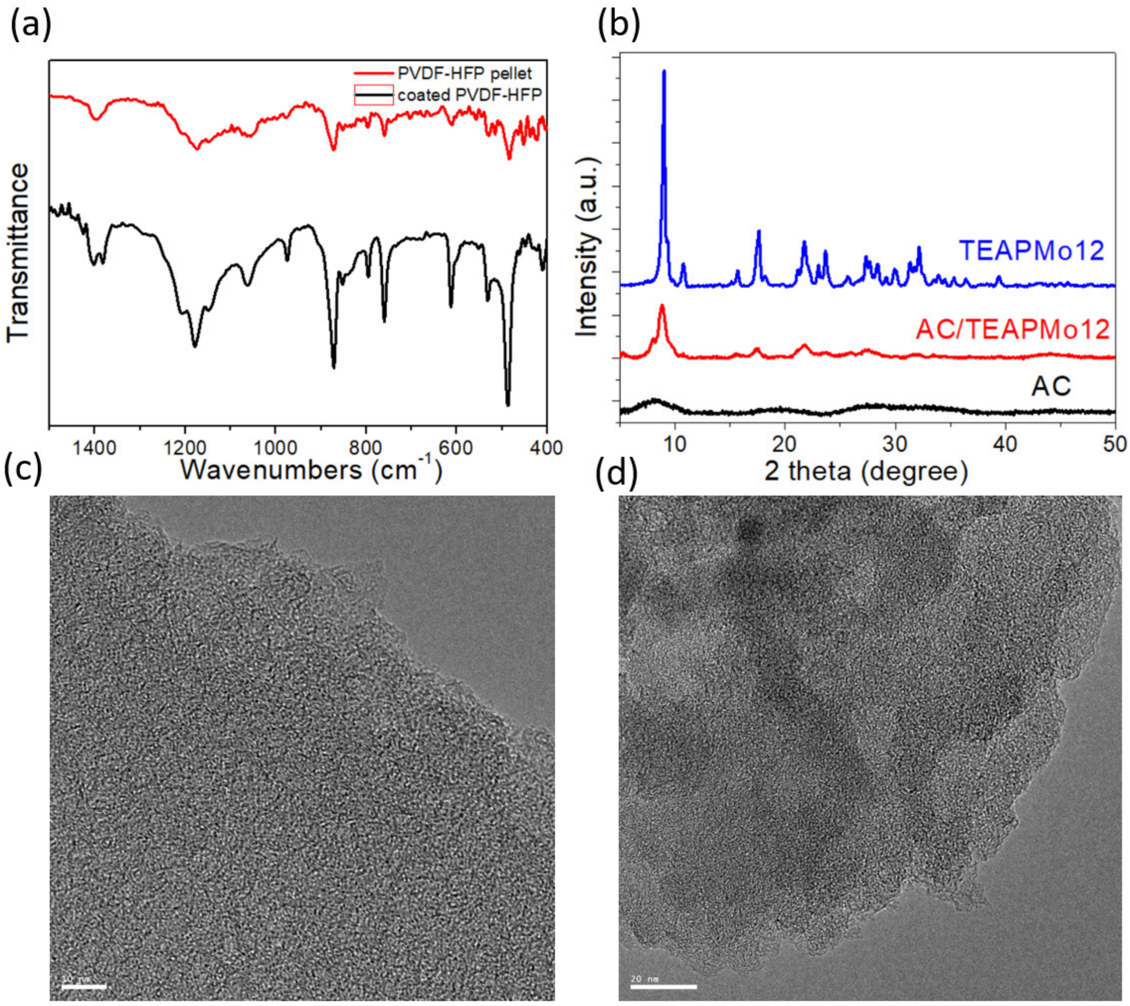

Figure S2. (a) FT-IR spectra of the pristine PVDF-HFP pellets and the as-prepared PVDF-HFP film. (b) XRD patterns of AC, AC/TEAPMo12 and TEAPMo12. TEM images of (c) AC and (d) AC/TEAPMo12.

Supplementary materials 3

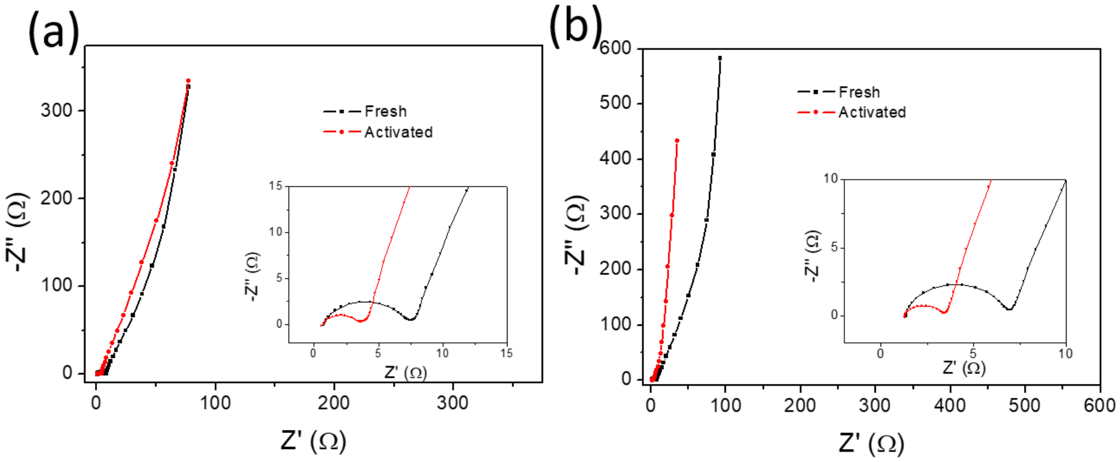

Figure S3. EIS spectra of AC symmetric (a) AC symmetric capacitor with PVDF binder/PVDF-HFP gel polymer electrolyte, (b) AC symmetric capacitor with PVDF-HFP binder/Cellulose separator/Liquid electrolyte

Table S3. the fitting results of impedance spectra.

Cells	state	R_s/Ω	C_{dl}/mF	R_{ct}/Ω	W_s-R/Ω	W_s-T/s	W_s-P
PVDF binder/PVDF-HFP gel polymer electrolyte	fresh	0.857	1.739	5.85	2.83	0.04	0.80
	cycled	0.744	2.512	2.58	1.72	0.03	0.84
PVDF-HFP binder/Cellulose separator/Liquid electrolyte	fresh	1.425	2.956	4.91	1.57	0.01	0.81
	cycled	1.364	5.291	1.79	0.76	0.01	0.86

Supplementary materials 4

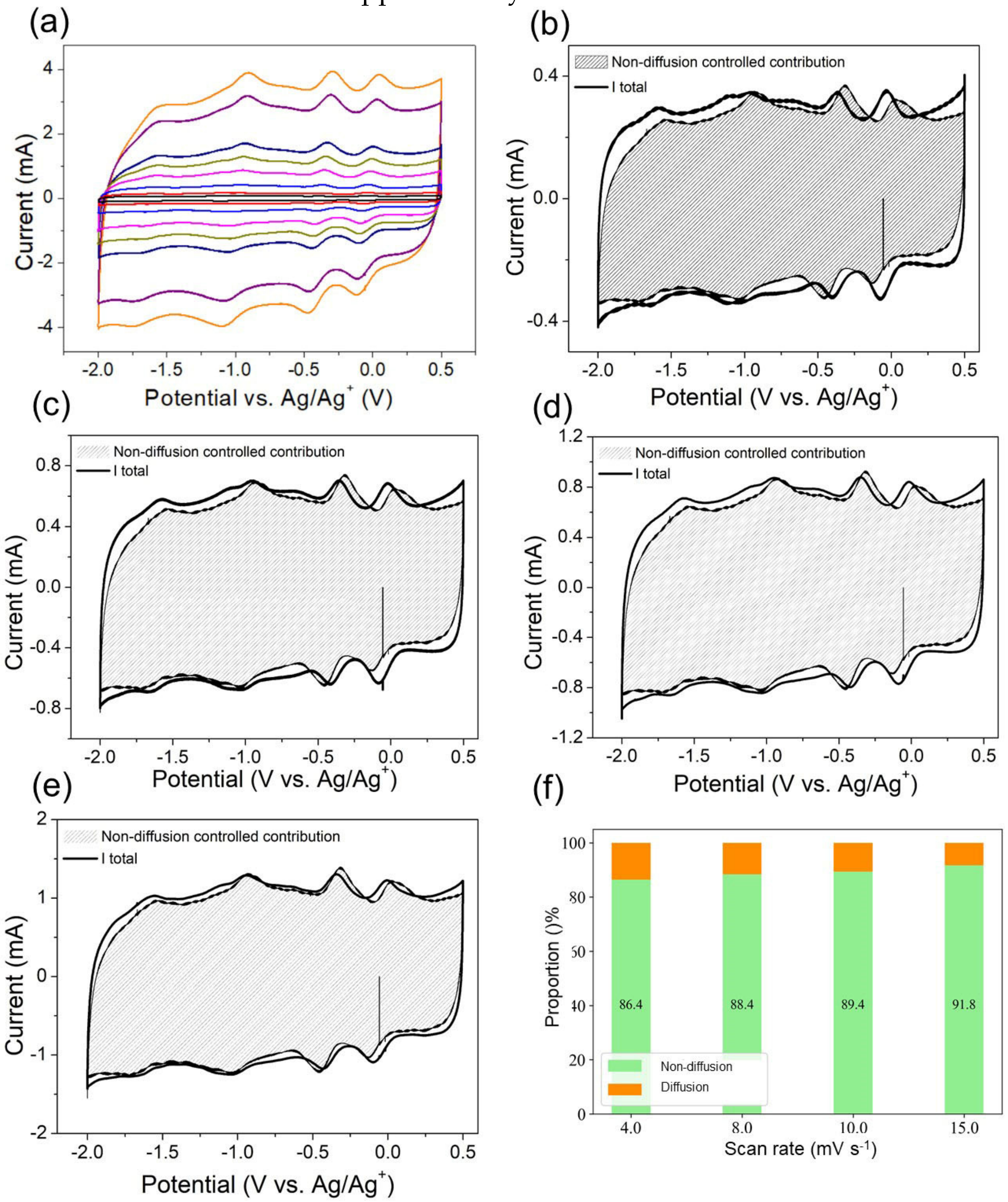

Figure S4. (a) CVs of AC/TEAPMo12 at various scan rates. Non-diffusion current vs. total current in CVs at (b) 4 mV s^{-1}, (c) 8 mV s^{-1}, (d) 10 mV s^{-1} and (e) 15 mV s^{-1}. (f) Non-diffusion-controlled process contribution vs. diffusion-controlled process contribution to the capacitance at various scan rates.

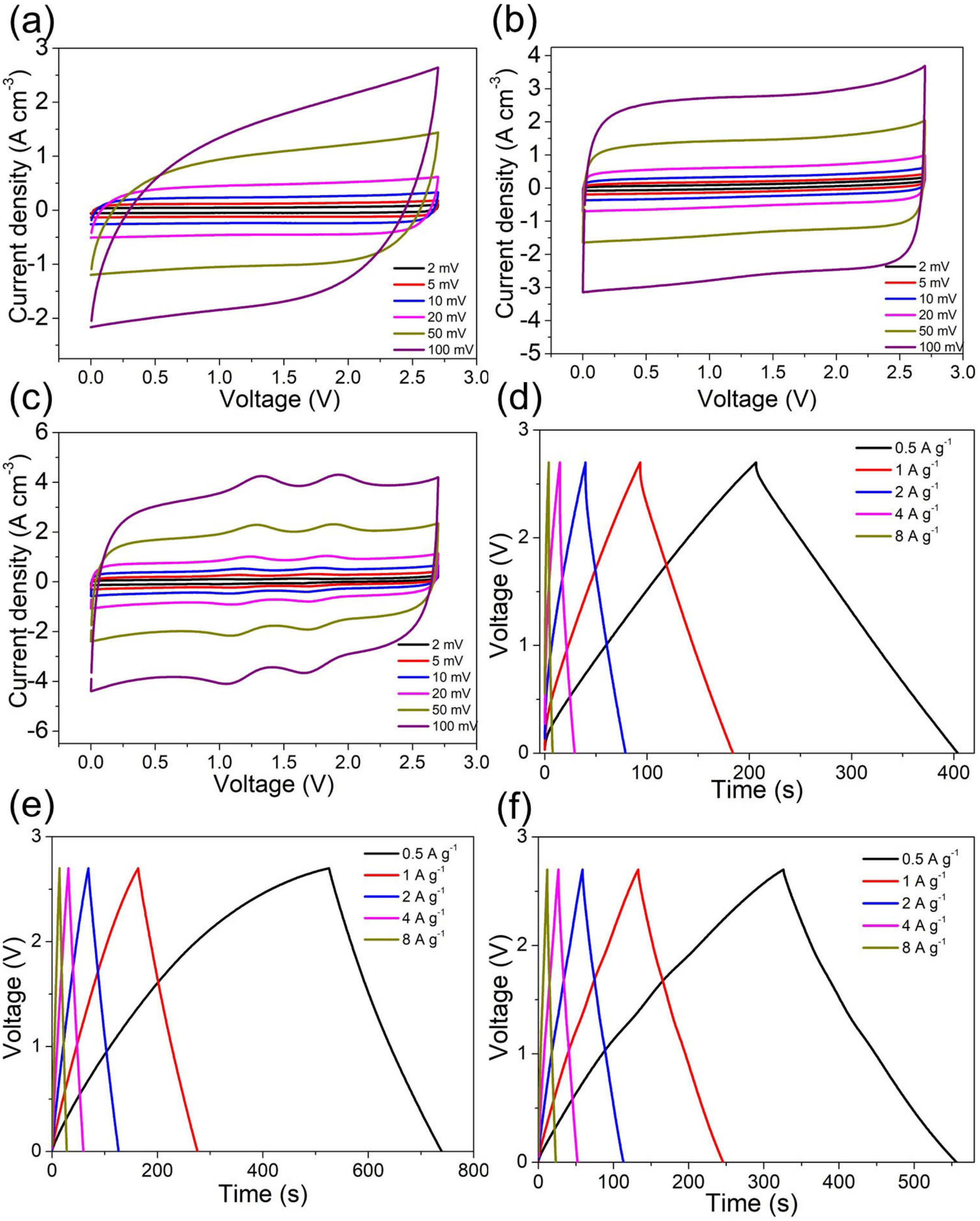

Figure S5. Cyclic polarization curves of (a) AC-Cellulose, (b) AC-GPE and (c) AC/TEAPMo12-GPE at various scan rates. Galvanostatic charging-discharging curves of (d) AC-Cellulose, (e) AC-GPE and (f) AC/TEAPMo12-GPE at various current densities.

Supplementary materials 6

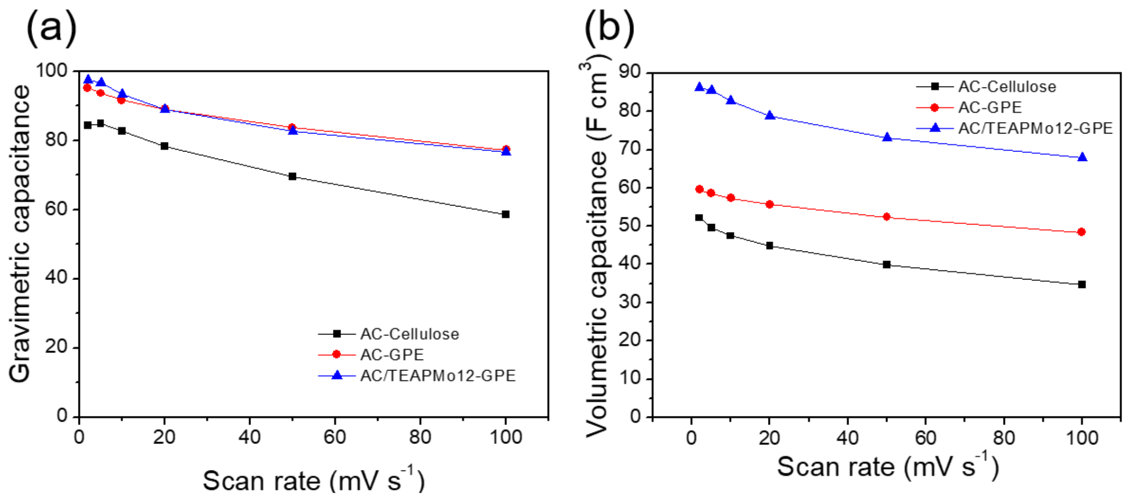

Figure S6. (a) Gravimetric and (b) volumetric capacitance of AC-Cellulose, AC-GPE and AC/TEAPMo12-GPE at various scan rates.
